# Higher PLIN5 but not PLIN3 content in isolated skeletal muscle mitochondria following acute in vivo contraction in rat hindlimb

**DOI:** 10.14814/phy2.12154

**Published:** 2014-10-15

**Authors:** Sofhia V. Ramos, Rebecca E. K. MacPherson, Patrick C. Turnbull, Kirsten N. Bott, Paul LeBlanc, Wendy E. Ward, Sandra J. Peters

**Affiliations:** 1 Department of Kinesiology, Brock University, 500 Glenridge Ave, St Catharines, L2S 3A1, Ontario, Canada; 2 Center for Bone and Muscle Health, Brock University, 500 Glenridge Ave, St Catharines, L2S 3A1, Ontario, Canada; 3 Department of Health Science, Brock University, 500 Glenridge Ave, St Catharines, L2S 3A1, Ontario, Canada

**Keywords:** Intramuscular triglycerides, lipolysis, OXPAT, perilipin family, TIP‐47

## Abstract

Contraction‐mediated lipolysis increases the association of lipid droplets and mitochondria, indicating an important role in the passage of fatty acids from lipid droplets to mitochondria in skeletal muscle. PLIN3 and PLIN5 are of particular interest to the lipid droplet–mitochondria interaction because PLIN3 is able to move about within cells and PLIN5 associates with skeletal muscle mitochondria. This study primarily investigated: 1) if PLIN3 is detected in skeletal muscle mitochondrial fraction; and 2) if mitochondrial protein content of PLIN3 and/or PLIN5 changes following stimulated contraction. A secondary aim was to determine if PLIN3 and PLIN5 associate and whether this changes following contraction. Male Long Evans rats (*n* = 21; age, 52 days; weight = 317 ± 6 g) underwent 30 min of hindlimb stimulation (10 msec impulses, 100 Hz/3 sec at 10–20 V; train duration 100 msec). Contraction induced a ~50% reduction in intramuscular lipid content measured by oil red‐O staining of red gastrocnemius muscle. Mitochondria were isolated from red gastrocnemius muscle by differential centrifugation and proteins were detected by western blotting. Mitochondrial PLIN5 content was ~1.6‐fold higher following 30 min of contraction and PLIN3 content was detected in the mitochondrial fraction, and unchanged following contraction. An association between PLIN3 and PLIN5 was observed and remained unaltered following contraction. PLIN5 may play a role in mitochondria during lipolysis, which is consistent with a role in facilitating/regulating mitochondrial fatty acid oxidation. PLIN3 and PLIN5 may be working together on the lipid droplet and mitochondria during contraction‐induced lipolysis.

## Abstract (precis)

This study investigates the mitochondrial enrichment of lipid droplet proteins, PLIN3 and PLIN5 in response to muscle contraction in rat hindlimb. Both PLIN3 and PLIN5 are detected in the mitochondrial fraction, but in response to 30 min electrically stimulated contraction designed to elicit muscle lipolysis, PLIN3 content does not change from resting values, but PLIN5 increased ~1.6 fold. This suggests an acute role for PLIN5 in mitochondria during contraction that has yet to be elucidated.

## Introduction

Lipid droplets are dynamic energy storage organelles, which function to store neutral lipids and are found in most tissue types (e.g., adipose tissue, skeletal muscle, and bone) (Murphy et al. 2009; Bosma et al. 2012a; Badin et al. 2013). Coated with a phospholipid monolayer, lipid droplets house triglycerides, sterol esters, and ceramides that can be used as substrates for the formation of intracellular membranes or energy production in the form of ATP (Prats et al. 2006; Murphy et al. 2009; Zehmer et al. 2009; Lass et al. 2011). Bound to and embedded in the lipid droplet membrane are the perilipin (PLIN1‐5) family of proteins, which are believed to be involved in lipid droplet formation and the breakdown of stored lipids (Greenberg et al. 1991; Londos et al. 1999; Robenek et al. 2005a; Kimmel et al. 2010). Of the PLIN family, PLIN3 and PLIN5 are found on the lipid droplet and in the cytosolic environment, interacting with other intracellular organelles and proteins (Wolins et al. 2001; Skinner et al. 2009; Badin et al. 2013; MacPherson et al. 2013a,b). The role of PLIN3 and PLIN5 in the liberation of fatty acids directly from lipid droplets is currently unknown. Further, it remains unknown if interactions of the PLINs with other intracellular organelles, such as mitochondria, are involved in lipid droplet breakdown and/or subsequent oxidation of the released fatty acids. In adipose tissue, fatty acids released from lipid droplets are sent into circulation, whereas in skeletal muscle, fatty acids are either taken up by mitochondria for oxidation or re‐esterified in the cytosol (Prats et al. 2006; Lafontan and Langin 2009). To this end, skeletal muscle lipid droplets are known to interact with mitochondria (Devries et al. 2007; Tarnopolsky et al. 2007; Shaw et al. 2008; Pu et al. 2011), but how this interaction occurs is poorly understood.

Endurance training in both male and female human subjects resulted in a decreased distance between lipid droplets and mitochondria, suggesting that the lipid droplet and mitochondria physically moved closer to each other. This is consistent with the increased reliance on intramuscular triglycerides as a fuel, typically seen posttraining (Mole et al. 1971; Hurley et al. 1986; Tarnopolsky et al. 2007). The association between lipid droplets and mitochondria is also observed following an acute bout of exercise in female participants who are known to rely heavily on fat oxidation after an acute bout of exercise (Devries et al. 2007). Thus, it appears that the lipid droplet and mitochondria in skeletal muscle work together or interact to promote efficient supply of fatty acids during acute and chronic exercise. However, the mechanism(s) governing this interaction and whether the proteins associated with the lipid droplet or mitochondria are involved is currently unknown.

PLIN5 is thought to play a role in fatty acid oxidation as it is predominantly expressed in tissues with a high oxidative capacity, such as skeletal muscle. Whole muscle overexpression of PLIN5 upregulates the expression of oxidative enzymes in skeletal muscle (Wolins et al. 2006; Bosma et al. 2013). More recently, PLIN5 protein content was detected in mitochondria from skeletal muscle and cardiac tissue (Yamaguchi et al. 2006; Bosma et al. 2012b). Mitochondria isolated from PLIN5 overexpressing rat tibialis anterior muscle did not augment mitochondrial density or respiration, but increased ^14^C‐palmitate oxidation suggesting that its function might not be involved with the electron transport chain and mitochondrial respiration but rather the fate of fatty acids when hydrolyzed (Wang et al. 2011; Bosma et al. 2012b). The novel finding of a lipid droplet associated protein, PLIN5, found within skeletal muscle mitochondria, is of special interest because with endurance training there is an increased lipid, mitochondrial, and PLIN5 content (Peters et al. 2012; Louche et al. 2013; Shepherd et al. 2013). Therefore, it is thought that PLIN5 may be a communicating link between the lipid droplet and mitochondria (Bosma et al. 2012b; Koves et al. 2013).

It is currently unknown if any other PLIN family proteins interact with skeletal muscle mitochondria, however, PLIN3 may be a likely candidate. PLIN3 was initially discovered by its involvement with the mannose‐6‐phosphate receptor, as an effector protein for Rab9 to the receptor complex to initiate intracellular vesicular movement (Diaz and Pfeffer 1998; Aivazian et al. 2006). Interestingly, in HeLa cells placed under oxidative stress, PLIN3 was recruited to the mitochondria and was able to stabilize the mitochondrial membrane preventing apoptosis (Hocsak et al. 2010b). While this intracellular function is still under investigation (Diaz and Pfeffer 1998; Barbero et al. 2001; Aivazian et al. 2006; Bulankina et al. 2009), this study exemplifies the ability of PLIN3 to move from the cytosol to the mitochondria under a specific perturbation such as the introduction of oxidative stress. What remains unknown is whether PLIN3 can be recruited to the mitochondria during stimulated lipolysis, as a function in skeletal muscle for this protein is currently unknown.

Previous work has identified interactions using coimmunoprecipitation methods, between PLIN3 and PLIN5 with ATGL, HSL, and CGI‐58 at rest, with no change in this interaction following contraction (MacPherson et al. 2013a,b; Mason et al. 2014). Moreover, PLIN3 and PLIN5 have been shown to inhabit the same smaller cytosolic lipid droplets in Chinese Hamster Ovary cells, potentially bringing those smaller lipid droplets to the larger lipid droplet pool or trafficking them for oxidation (Bartholomew et al. 2012). During rest, contraction to induce lipolysis, epinephrine or the combination of contraction and epinephrine, PLIN3 and PLIN5 appear to be serine phosphorylated (MacPherson et al. 2013a,b), therefore leaving the mechanism(s) of regulation unknown. Due to the above‐mentioned similarities between PLIN3 and PLIN5 it is possible that these two PLIN proteins are interacting with each other. Whether this interaction exists and changes with contraction to induce lipolysis is currently unknown.

Research thus far points toward a potential role for PLIN3 and PLIN5 in contraction‐mediated lipolysis in skeletal muscle, particularly with respect to a role in the mitochondria. The main purpose of this study was a) to determine if PLIN3 protein is present in skeletal muscle mitochondria; and b) to determine any change in PLIN3 or PLIN5 protein content in skeletal muscle mitochondria following a 30 min in vivo electrically stimulated contraction period to elicit lipolysis (Stefanyk et al. 2012). We hypothesize that PLIN3 will be present in rested skeletal muscle mitochondria and that the mitochondrial protein content of both PLIN3 and PLIN5 will increase with stimulated contraction. A secondary purpose of this study is to determine if an interaction exists between PLIN3 and PLIN5 and whether this interaction changes following contraction. We hypothesize that this interaction will not change as seen with the other reported interactions between these PLIN proteins and skeletal muscle lipases and coactivators (MacPherson et al. 2013a,b).

## Methods

### Animals

Male Long‐Evans rats (*n* = 21, body weight = 317 ± 6 g) at approximately 3 months of age were used for this study. Rats were purchased from Charles River Laboratories (Canada). All procedures and protocols were approved by the Animal Care and Utilization Committee at Brock University and conform to all Canadian Council on Animal Care guidelines (Olfert et al. 1993). Rats were housed in pairs in the Comparative Bioscience Facility, maintained on a 12:12 light–dark cycle, fed standard rodent chow, Tekland Global 14% protein, (Harlan Tekland Global, Mississauga, ON, Canada) and had ad libitum access to food and water.

### Sciatic nerve stimulation protocol

Anaesthetized rats underwent sciatic nerve stimulation for 30 min (10 msec impulses, 100 Hz/3 sec at 10–20 V; train duration 100 msec), which has previously been shown to induce lipolysis in rat hindlimb muscles (Stefanyk et al. 2012). This protocol consists of two 13 min halves with a 4 min break in between. Rats were anaesthetized with sodium pentobarbital (6 mg/100 g of body weight) via intraperitoneal injection. A small incision was made on the left leg above the hip to expose the sciatic nerve. Curved platinum electrical wires were attached to the sciatic nerve for stimulation with the left leg being stimulated, whereas the right remained as a resting control. Upon completion of the stimulated contraction, the plantaris muscle (*n* = 5) was removed and prepared for mechanical sarcolemma isolation (Fajardo et al. 2013). Red gastrocnemius muscles were then excised and divided as follows; the belly of the muscle was cut and set in embedding compound (Cryomatrix, Pittsburgh, PA) and cooled in methyl‐butane for 90 sec before being stored at −80°C. From the remaining red gastrocnemius, approximately 30 mg was cut off (*n* = 9/group) and snap frozen for protein analysis, whereas the remaining muscle (*n* = 15/group) was prepared for primarily subsarcolemmal mitochondria isolation.

### Skeletal muscle lipid staining

Embedded red gastrocnemius muscles (*n* = 11/group) were cut into transverse sections using a cryotome (ThermoShandon, Runcorn, Cheshire, UK) set at −20°C, cutting 10‐*μ*m‐thick sections, and then mounted on microscope slides. Microscope slides containing muscle sections were fixed in 3.7% formaldehyde for 1 h followed by a 30 min immersion in a diluted working solution of oil red‐O (oil red‐O; O0625; Sigma‐Aldrich, St. Louis, MO) as described in Koopman et al. (2001) and previously done in our lab (MacPherson et al. 2012, 2013a,b). Slides were then washed three times, 5 min per wash, with deionized water. Once dried, each muscle section was coated with 10 *μ*L of anti‐fade reagent (no. P36930; Prolong Gold Anti‐fade Reagent; Invitrogen, Burlington, ON, Canada) and covered with a glass cover slip. Slides were stored in the dark overnight to dry.

### Imaging and analysis

Muscle sections were analyzed using a Nikon Eclipse 80i fluorescence microscope (Nikon Eclipse 80i; Chiyoda‐ku, Tokyo, Japan) and images were captured with a digital camera attached to the microscope (Retiga 1300, QImaging, Burnaby, BC, Canada). Oil red‐O stain was visualized with 550 fluorophore. Images were captured at 40× magnification, with three fields of view/muscle cross section (18.8 ± 0.98 fibers/field of view). Each fiber (57 ± 3.00 fibers/sample) was manually outlined to determine lipid content using imaging software (NIS‐Elements AR 3.00; Nikon Instruments, Melville, NY). Lipid content was quantified by manually selecting an intensity threshold that was applied to all images. The number, area, and objects within the fibers emitting a fluorescent signal were recorded and expressed as the fraction of the measured area that was stained.

### Subsarcolemmal mitochondrial isolation and purification

The mitochondria isolation protocol described has been adapted from previous methods and used in our lab (Jackman and Willis 1996; Peters et al. 2001; Stefanyk et al. 2010). Briefly, fresh muscles were directly placed on an inverted glass plate, on ice and manually minced. Samples were then immersed in 20 times the volume of solution 1 (100 mmol/L KCl, 40 mmol/L Tris HCl, 10 mmol/L Tris base, 5 mmol/L MgSO_4_, 5 mmol/L EDTA and 1 mmol/L ATP) and manual homogenized with a glass homogenizer. Samples underwent differential centrifugation; homogenate was centrifuged for 10 min at 700 g, and supernatant was collected and spun at 14,000 g to extract mitochondria. Mitochondria were then resuspended in 10 times the volume of solution 2 (100 mmol/L KCl, 40 mmol/L Tris HCl, 5 mmol/L Tris base, 1 mmol/L MgSO_4_, 0.01 mmol/L EDTA, 1% BSA, and 0.25 mmol/L ATP) and washed for 10 min at 7000 g followed by a wash in solution 3 (100 mmol/L KCl, 40 mmol/L Tris HCl, 5 mmol/L Tris base, 1 mmol/L MgSO_4_, 0.01 mmol/L EDTA, and 0.25 mmol/L ATP) for 10 min at 7000 g. Mitochondria were further purified with a 60% Percoll ^®^ (P1644, Saint Louis, MO) gradient and resuspended in sucrose and mannitol solution (220 mmol/L sucrose, 70 mmol/L mannitol, 10 mmol/L Tris HCl, and 0.1 mmol/L EDTA). Samples were stored at −80°C until protein analysis.

The purity of the mitochondrial fraction was determined by western blotting. Contamination for sarco (endo) plasmic reticulum was measured by SERCA1 and SERCA 2 protein content, lipid droplet contamination was assessed by PLIN2 protein content, and sarcolemma contamination was measured by beta‐dystroglycan protein content. All membranes were cut at ~25 kDa band and probed for COX IV (10 kDa) to confirm the presence of mitochondria and used as a loading control.

### Mechanical sarcolemma isolation

As a precaution, sarcolemmal cuffs were collected from the plantaris muscle to determine if PLIN3 and/or PLIN5 content were present in the sarcolemma and/or if it changed with contraction as seen with PLIN1B and insulin stimulation in human adipocytes (Aboulaich et al. 2006). Sarcolemmal cuffs were isolated as previously described (Fajardo et al. 2013). Briefly, fibers were isolated using microdissecting forceps and visualized with a dissecting microscope (Nikon SMZ645 with a Nikon 3002752 objective and Nikon C‐W10 9 A/22 eyepiece). Plantaris muscles were placed in a petri dish at room temperature suspended in Sylgard with a resting solution (Sylgard 184, DOW Corning) (90 mmol/L 4(2‐hydroxyethyl)‐1‐piperazineethanesulfonic acid, 50 mmol/L ethylene glycol tetraacetic acid, 10.3 mmol/L magnesium oxide, 8 mmol/L ATP, 10 mmol/L creatine phosphate, pH 7.1 with 4 mol/L sodium hydroxide). A group of fibers were pulled away from the muscle belly, teasing out individual fibers. Individual fibers were then grabbed and pulled at each end, splitting it apart until the sarcolemma rolled back on its self, forming a cuff. For each cuff collected, the respective skinned fiber was collected. Ten of each cuff, skinned fiber, and whole fiber were isolated and stored in 20 *μ*L of membrane preserving buffer (10 mmol/L NaHCO_3_, 0.25 mol/L sucrose) at −80°C until protein analysis. For western blotting, 10 *μ*L of 3× Laemmli buffer was added to each micro centrifuge tube containing sarcolemmal cuffs, skinned fibers, and whole fibers followed by three freeze–thaw cycles. Samples were then homogenized with a micro centrifuge tube plunger and spun down before loading.

### Antibodies

The antibodies used for this study have been previously used in our laboratory (MacPherson et al. 2012, 2013a; Fajardo et al. 2013) and other laboratories (Carroll et al. 2001; Robenek et al. 2005b; Aivazian et al. 2006; Bosma et al. 2013) and are as follows: PLIN3, (Anti‐Tip47 (NT) rabbit polyclonal, ProSci Incorporated, #3883, CA), PLIN2 (Adipophilin/ADRP mouse monoclonal, Progen, #610102, Heidlberg, Germany), PLIN5 (Anti‐OXPAT, guinea pig polyclonal, Progen, #GP31, Heidlberg, Germany), cytochrome c oxidase complex 4 (COX IV) (COX4 subunit 4, mouse monoclonal, MitoScience, #MS407, TO), Beta‐dystroglycan (mouse monoclonal, Abcam, #ab49515, Cambridge, MA), SERCA1 (Anti‐SERCA1 ATPase, mouse monoclonal, Thermoscientific, #MA3‐911, Rockford, IL), and SERCA2 (Anti‐SERCA2 ATPase, mouse monoclonal, Abcam, #ab2861, Cambridge, MA).

### Western blotting

Protein concentration was determined by Bradford assay (Bio‐Rad Protein Assay Dye Reagent Concentrate; #500‐0006; Bio‐Rad, Mississauga, ON, Canada); red gastrocnemius immunoprecipitated muscle (*n* = 9) and mitochondria samples (*n* = 15) were prepared with a 3× Laemmli buffer and boiled for 5 min (except for PLIN5) before loading. An 8% (PLIN3, (8*****μ*g)) or 10% (PLIN2 (15 *μ*g), PLIN5 (30 *μ*g) SERCA1 (20 *μ*g), SERCA2 (10 *μ*g), and beta‐dystroglycan (10 *μ*g)) gel were made for protein separation for ~80 min at 120V. Proteins were transferred onto polyvinlidene difluride membranes at 100 V for 60 min. All membranes were cut at the ~25 kDa marker and probed for COX IV (10 kDa) to quantify as a loading control. Membranes were blocked with 5% fat‐free milk (PLIN2, PLIN5, SERCA1, SERCA2, beta‐dystroglycan, and COX IV) or 5% bovine serum albumin (BSA) (PLIN3) and diluted with the appropriate primary antibody (1:1000 for PLIN2, PLIN3, PLIN5, SERCA1, SERCA2; 1:500 for beta‐dystroglycan; 2:5000 for COX IV) with 5% milk (COX IV), 3% milk (PLIN2, PLIN5, SERCA1, SERCA2, beta‐dystroglycan) or 1% BSA (PLIN3) overnight. All membranes were washed in TBST three times for 5 min and then incubated with secondary antibodies conjugated with horseradish peroxidase (1:10,000 for all proteins except for COX IV which was 1:20,000) diluted in 5% milk (COX IV), 3% milk (PLIN2, PLIN5, SERCA1, SERCA2, beta‐dystroglycan) or 1% BSA (PLIN3) for 1 h. All membranes were washed with TBST three times for 10 min and visualized with Chemiluminescent horseradish peroxidase reagent substrate (Peroxide solution + Luminol reagent) (Amersham Biosciences, Piscataway, NJ). Images were analyzed using Image J software (http://rsbweb.nih.gov/ij/).

### Determination of PLIN3–PLIN5 protein interaction

Snap frozen red gastrocnemius muscles (*n* = 9/group) were homogenized with 20 times the volume of Griffin lysis buffer (150 mmol/L NaCl, 50 mmol/L Tris HCl, 1 mmol/L EGTA) with protease (1183617001; Roche Diagnostics, Lava, OC, Canada) and phosphatase (0.406845001; Roche Diagnostics, Lava, QC, Canada) inhibitor tablets. Protein concentration was determined using a Bradford Assay. 1000 *μ*g of protein from muscle homogenates were suspended in covalent resin‐to‐antibody complexes made with Pierce Co‐Immunoprecipitation (Co‐IP) kit (Thermo Scientific, #26149) and PLIN5 primary anti‐body to immobilize PLIN5 protein. Each sample underwent western blotting as described above to confirm PLIN5 protein (~52 kDa) was immunoprecipitated loading 20 *μ*g of protein from the immuoprecipitated samples and 30 *μ*g of protein loaded from red gastrocnemius whole homogenates. Each membrane was then stripped and reprobed with Restore^™^ Plus Western blot stripping buffer (Thermo Scientific, #46430) and probed for PLIN3 (47 kDa). PLIN3 content was made relative to precipitated PLIN5 content.

### Statistical analysis

Total lipid content was measured in rested and contracted samples with a paired one‐tailed t‐test. Protein content in mitochondria extracted from rest and contracted muscles were normalized to amount of COX IV loaded per lane and analyzed with a paired one‐tailed t‐test (GraphPad Prism; La Jolla, CA). PLIN5‐to‐PLIN3 interaction was normalized to precipitated PLIN5 protein with a two‐tailed paired *t*‐test. Western blot band density was analyzed with ImageJ software (http://rsbweb.nih.gov/ij.). Statistical significance for all tests was accepted at *P* < 0.05.

## Results

### Neutral lipid utilization during electrically stimulated contraction

**Figure 1.**
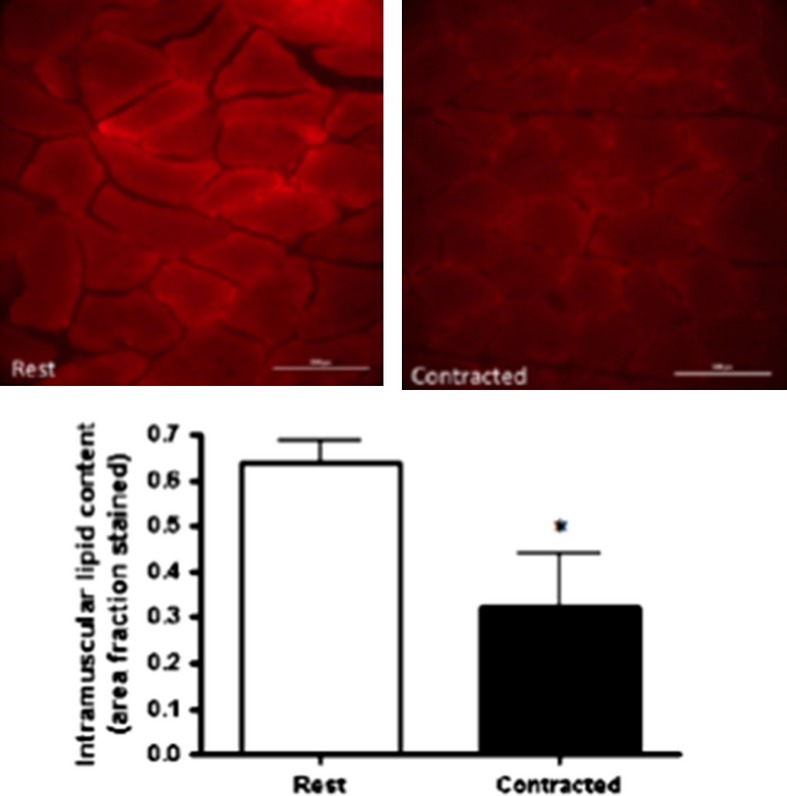
(A) Oil red O immunofluorescent staining of rest and contracted red gastrocnemius muscle (*n* = 11). Images of one single field of view of muscle cross section. (B) Lipid droplet content (area fraction stained) at rest and following stimulated contraction. Values are expressed as mean ± standard error. Lipid droplet content is significantly lower in contracted muscles when compared with rested muscle (*P* = 0.03). *Denotes significance accepted at (*P* > 0.05).

Stimulated contraction caused ~50% reduction in intramuscular lipid content when compared with rested red gastrocnemius muscle (rest, 0.63 ± 0.04% area lipid stained; contracted, 0.32 ± 0.11% area lipid stained, *P* = 0.03) (Fig. 1).

### Mitochondria contamination

**Figure 2.**
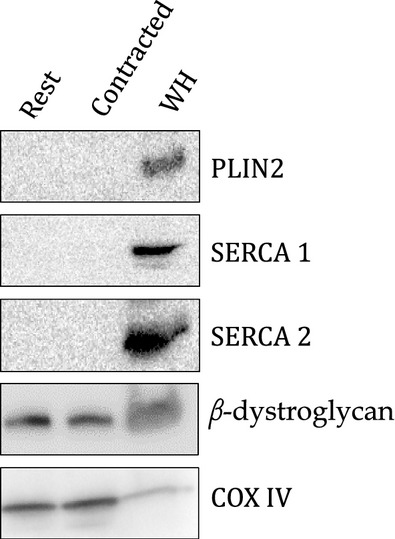
Representative western blot of rested and contracted mitochondria samples probing for proteins from different intracellular organelles as follows (*n* = 15); loading 15 *μ*g of mitochondrial protein for PLIN2 (lipid droplets), 20 *μ*g of mitochondrial protein for SERCA 1 and 10 *μ*g of mitochondrial protein for SERCA 2 (endo(sarco)plasmic reticulum) and 10 *μ*g of mitochondrial protein for beta‐dystroglycan (sarcolemma) and whole homogenate (WH) with COX IV from corresponding membranes. PLIN2, SERCA1 and SERCA 2 are undetectable in mitochondria samples loaded. Beta‐dystroglycan was detected in purified mitochondria samples with no significant differences between rest and contraction (*P* = 0.62).

**Figure 3.**
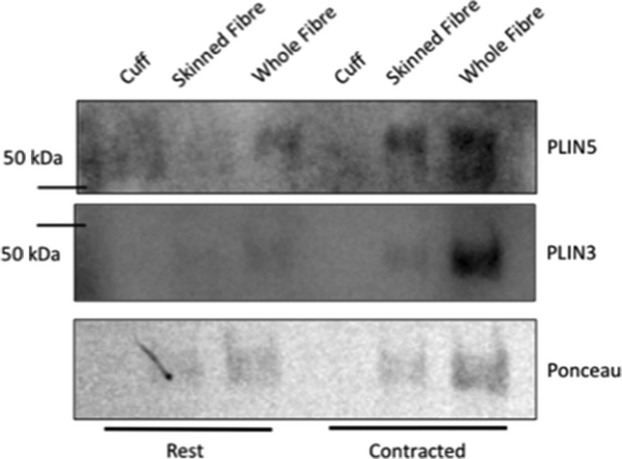
Representative western blot of sarcolemma cuffs (four cuffs loaded), skinned fiber (four skinned fibers loaded) and whole fiber (four whole fibers loaded) of rested and contracted plantaris muscle (*n* = 5) to confirm that PLIN3 and PLIN5 are not detectable. (A) Sarcolemmal cuff, skinned fiber, and whole fiber probed PLIN5. (B) Sarcolemmal cuff, skinned fiber, and whole fiber probed for PLIN3.

There was no detectable contamination of lipid droplets (PLIN2) or sarco(endo)plasmic reticulum (SERCA1 and SERCA2) in purified red gastrocnemius mitochondrial samples (Fig. 2). Contrary to the literature (Campbell et al. 2004; Yoshida et al. 2007; Stefanyk et al. 2010; Hoshino et al. 2013), there was some sarcolemmal contamination (beta‐dystroglycan) present in the purified red gastrocnemius mitochondria with no significant difference from rest to contraction (*P* = 0.62). However, PLIN3 and PLIN5 were not found in plantaris sarcolemmal cuffs (Fig. 3), nor was there enrichment in the sarcolemma with contraction.

### PLIN5 protein content in mitochondria

**Figure 4.**
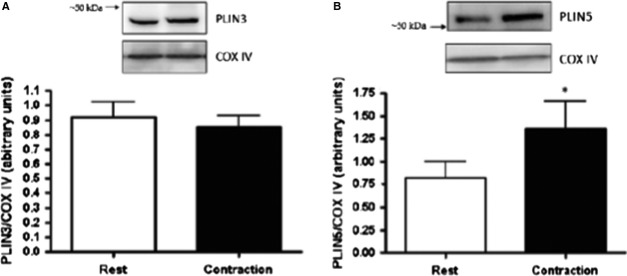
PLIN3 (A) and PLIN5 (B) protein content in rested and contracted skeletal muscle mitochondria (*n* = 15). (A) Representative western blot of PLIN3 at rest; *lane 1 (8 μ*g of protein loaded) and following stimulated contraction*; lane 2*. No significant difference in PLIN3 association with isolated mitochondria following contraction (*P* = 0.21). (B) Representative western blot of PLIN5 (30 *μ*g of protein loaded) at rest; *lane 1* and following contraction; *lane 2*. 64% increase in PLIN5 content in isolated mitochondria following contraction (*P* = 0.009).

PLIN5 content was ~1.6‐fold higher in mitochondria isolated from the stimulated versus unstimulated red gastrocnemius mitochondria (*P *= 0.009) (Fig 4).

### PLIN3 protein content in mitochondria

With contraction‐mediated lipolysis, red gastrocnemius mitochondrial PLIN3 protein content was unchanged (*P* = 0.21) (Fig. 4).

### PLIN3 and PLIN5 whole muscle protein content

**Figure 5.**
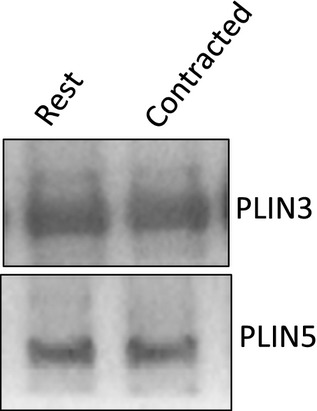
Representative western blot of total homogenate PLIN3 and PLIN5 in red gastrocnemius whole muscle *(PLIN3; 10 μ*g of protein loaded, PLIN5; 30 *μ*g of protein loaded) at rest; *lane 1* and following contraction; *lane 2*.

In red gastrocnemius whole muscle homogenates, PLIN3 and PLIN5 protein content was unaltered following stimulated contraction (Fig. 5).

### PLIN3 and PLIN5 protein–protein interaction

**Figure 6.**
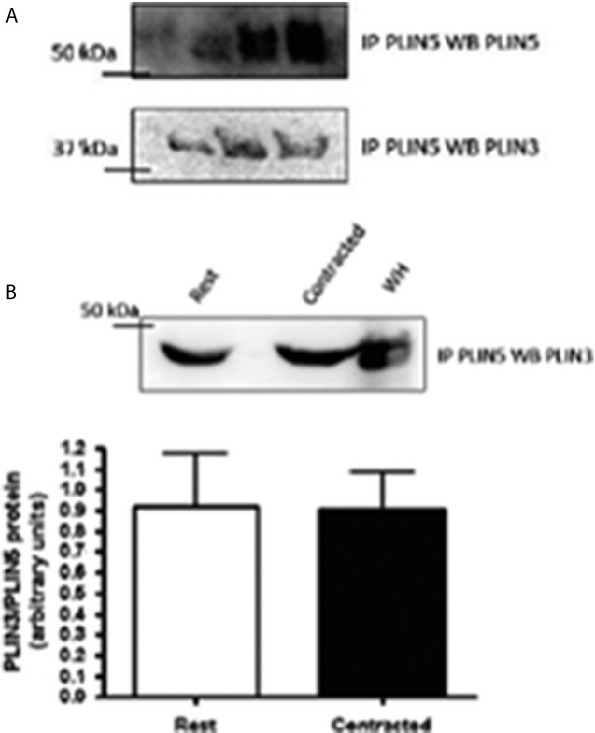
Representative western blots of the interaction between PLIN3 and PLIN5 (*n* = 9). (A) Precipitated PLIN5 from red gastrocnemius whole homogenate samples, western blotted for PLIN5 showing up at approximately 52 kDa. Membrane from stripped and reprobed for PLIN3 showing up at approximately 47 kDa. (B) Representative western blot of immunoprecipitate PLIN5 samples probed for PLIN3. Graph depicting no change in the interaction between PLIN3 and PLIN5 at rest and following contraction (*P* = 0.65). IP: immunoprecipitate, WB: western blot, WH: whole homogenate.

A PLIN3–PLIN5 interaction was measured at rest in whole red gastrocnemius homogenate. This interaction did not change following stimulated contraction (*P* = 0.65) (Fig. 6).

## Discussion

This study is the first to demonstrate that mitochondrial PLIN5 content in skeletal muscle is increased after 30 min of electrically stimulated contraction that induced lipolysis. As PLIN5 is higher in the mitochondrial fraction with contraction, it is consistent with a role for PLIN5 in coordinating the release of fatty acids from the lipid droplet and their subsequent oxidation in the mitochondria (Wang et al. 2011; Bosma et al. 2013; Koves et al. 2013). Other novel findings are that PLIN3 protein can be detected in skeletal muscle mitochondria, and the net mitochondrial PLIN3 protein content remained unchanged following contraction. Evidence from the literature would suggest that PLIN3 and PLIN5 are important players in skeletal muscle lipid droplet metabolism (Bartholomew et al. 2012; MacPherson et al. 2013a,b). Our data add to the emerging story that PLIN3 and PLIN5 appear to have a role by associating with not only lipid droplets, but also with mitochondria.

The in vivo sciatic nerve stimulation model used in this study provided the opportunity to pair each contracted muscle to its own resting control, in addition to maintaining physiological conditions during stimulation. This stimulation protocol has been previously used to elicit intracellular fatty acid mobilization (Han et al. 2007; Stefanyk et al. 2012) although this is the first study to actually report that there is an almost 50% reduction in the amount of muscle lipids following contraction. This confirms that this is an appropriate model to study muscle lipolysis for energy provision during contraction. This reduction in muscle lipid content corroborates other studies using in vivo sciatic nerve and isolated soleus stimulation (Spriet et al. 1986; Dyck and Bonen 1998; MacPherson et al. 2012, 2013a).

### PLIN5 and PLIN3 protein in skeletal muscle mitochondrial extracts

Our study is the first to demonstrate PLIN5 enrichment in skeletal muscle mitochondria following 30 min of in vivo stimulated contraction. This suggests a role for PLIN5 in the lipid droplet‐to‐mitochondria communication that occurs during contraction that induces lipolysis. PLIN5 is widely known for its involvement in fat storage and oxidation as its tissue expression is positively correlated with tissue oxidative capacity (Wolins et al. 2006; Minnaard et al. 2009; Peters et al. 2012). Unlike adipose tissue, fatty acids released from skeletal muscle lipid droplets are shuttled directly to mitochondria for oxidation (Horowitz et al. 2000; Horowitz 2003). Our results suggest that PLIN5 is perhaps involved in the process of shuttling these fatty acids to the mitochondria due to the robust increase in mitochondrial PLIN5 content after only 30 min of stimulated contraction. More recently, Mason and colleagues (Mason et al. 2014) analyzed the colocalization between PLIN5 and the mitochondria in human vastus lateralis muscle following 60 min of moderate intensity exercise. Here, they were unable to detect an increased colocalization between PLIN5 and the mitochondria in response to their exercise perturbation which may be explained by an unchanged intramuscular lipid content measured by oil red‐O in this model. Our contraction protocol elicited an approximate 50% reduction in neutral lipids demonstrating that a large amount of triglycerides were broken down which would then require a chaperone or conduit to bring them to the mitochondria for oxidation.

Shepherd et al. (2013) demonstrated that lipid droplets coated with PLIN5 were preferentially used over lipid droplets not coated with PLIN5 in vastus lateralis collected from sedentary males following both sprint interval and endurance training. Although the exact intracellular location of PLIN5 is not clear, we assume that PLIN5 is chaperoning or directing fatty acids liberated from lipid droplets to the mitochondria during energy requiring situations. Red gastrocnemius whole muscle results indicate no change in PLIN5 protein content at rest and following acute contraction which would suggest a reorganization or movement of intracellular PLIN5 protein in response to lipolysis stimulated by contraction. PLIN5 protein is not quantifiable in tissues with low mitochondria content (e.g., adipose tissue) (Wolins et al. 2006; Yamaguchi et al. 2006; Dalen et al. 2007; Bickel et al. 2009) and is most abundant PLIN in tissue that heavily relies on fat oxidation (e.g., cardiac and skeletal muscle), which is consistent with the proposed function for PLIN5 (Wang et al. 2011; Bosma et al. 2012b, 2013). Further research is needed to locate the exact intracellular location of PLIN5 to determine if this mitochondrial enrichment is due to involvement of PLIN5 in regulating the fate of fatty acids during contraction, whether they are hydrolyzed from lipid droplets or recruited from the cytosol to the mitochondria.

This is the first study to indicate that PLIN3 is also found in skeletal muscle mitochondria, and contrary to our hypothesis, this content was unaltered with muscle contraction that induced lipolysis. Due to its ubiquitous expression throughout various tissues (Wolins et al. 2006) and interactions with intracellular organelles (Skinner et al. 2009; Hocsak et al. 2010a), PLIN3 is thought to have a variety of functions within many different cell types, including; lipogenesis, lipolysis, and trafficking (Wolins et al. 2001; Aivazian et al. 2006; Buers et al. 2009; Skinner et al. 2009; Hocsak et al. 2010a; MacPherson et al. 2013a). A discrete function for PLIN3 in skeletal muscle has yet to be determined. Prats et al. (2006) measured no net change in PLIN3 localization to the lipid droplet with lipolytic stimulation in solei from male Wistar rats, and our results also show no net change in PLIN3 content in red gastrocnemius mitochondria. From this data, we can speculate that PLIN3 may act as a type of trafficking protein, regulating the placement of proteins and/or organelle in specific position within the cell and moving them when needed. Therefore, this would require a certain amount of PLIN3 to be present on the lipid droplet and mitochondria to mediate this interaction. This would also be consistent with the proposed role of PLIN3 in intracellular trafficking and the mannose‐6‐receptor. Here, PLIN3 functions as an effector protein for Rab9‐GTPase, recruiting this trafficking protein to the receptor complex where it can initiate the movement of the mannose‐6‐receptor containing vesicles throughout the cell (Diaz and Pfeffer 1998; Barbero et al. 2002; Aivazian et al. 2006). It is thought that with an acute moderate intensity exercise bout and endurance training, skeletal muscle relies on fatty acids for fuel (Davies et al. 1981). In addition, after acute exercise and endurance training the association between mitochondria and lipid droplets significantly increases (Devries et al. 2007; Tarnopolsky et al. 2007; Amati et al. 2011). PLIN3 may be a protein involved in mediating this interaction as it was originally discovered being involved in vesicular transportation (Diaz and Pfeffer 1998; Aivazian et al. 2006). The potential mechanism of action requires further exploration and the potential involvement of Rab proteins that associate with lipid droplets and mitochondria.

### PLIN3–PLIN5 protein interaction

In addition to the novel findings of the involvement of PLIN3 and PLIN5 with the mitochondria, using co‐immunoprecipitation methods, we found an interaction between PLIN3 and PLIN5. As expected, this interaction did not change following 30 min of stimulated contraction. While we cannot rule out the possibility of a protein–lipid–protein interaction involving PLIN3 and PLIN5 as suggested previously (Bartholomew et al. 2012), these two proteins might be working together, as all lipases and proteins seem to be in close proximity to each other in skeletal muscle. We observed no net change in PLIN3 content in mitochondria, but we cannot discount the possibility that PLIN3 is moving back and forth from the lipid droplet to the mitochondria, leaving a consistent amount of PLIN3 at the mitochondria. Previous work from our laboratory identified protein–protein interactions with PLIN3 and PLIN5 individually with CGI‐58, ATGL, and HSL under rest, stimulated contraction, epinephrine stimulation, and the combination of epinephrine and contraction in skeletal muscle (MacPherson et al. 2013a,b). It is likely that these PLIN proteins work together in a complex form to regulate lipid droplet dynamics.

### Mitochondrial purity and sarcolemmal cuff analysis

Using a large lower limb that is mixed and oxidative (red gastrocnemius; predominately type I: 51% population, and IIA: 35% population) (Delp and Duan 1996) allowed us to perform all three protein and lipid analysis using the same muscle. Thus, our experimental approach served to minimize individual variation among rats and to focus on a single muscle for all measurements, except for analysis of sarcolemmal cuffs. Sarcolemmal cuffs were collected from the plantaris muscle because it is a fusiform muscle that allows us to perform the mechanical isolation of the cuffs. The soleus and extensor digitorum longus muscles are also fusiform muscles, but the fiber‐type populations are at the two extremes (soleus; predominately type I: 84% population and extensor digitorum longus; predominantly type IID/X: 38% and IIB: 38% population) (Delp and Duan 1996), which would make it difficult to interpret the results to a more mixed fiber type like the red gastrocnemius muscle. However, as it is a different muscle, we cannot exclude the possibility of enrichment of PLIN3 and/or PLIN5 in the red gastrocnemius sarcolemma following contraction.

To ensure that the mitochondrial extracts were free from contamination of other intracellular structures, samples underwent a Percoll^®^ gradient for purification and probed for markers of intracellular organelles. Our results suggest that there is no contamination from lipid droplets and sarco(endo)plasmic reticulum which might be expected to contain either PLIN3 or PLIN5, as they have been shown to interact with other intracellular structures in cell culture models (Skinner et al. 2009; Hocsak et al. 2010b; Bartholomew et al. 2012). There was a measurable contamination of a sarcolemmal marker, beta‐dystroglycan, in our purified mitochondria, which is contrary to previous work using the Na^+^/K^+^ ATPase as a marker of sarcolemmal contamination (Campbell et al. 2004; Yoshida et al. 2007; Stefanyk et al. 2010; Hoshino et al. 2013). Primarily subsarcolemmal mitochondria were isolated for these experiments using differential centrifugation, which might not be the most gentle method of isolation, so we might expect to have sarcolemmal contamination as the mitochondria and sarcolemma are in close proximity in skeletal muscle (Dombrowski et al. 1996; Bloch and Gonzalez‐Serratos 2003; Shaw et al. 2008). In addition, an appropriate marker for sarcolemma has yet to be identified (Fajardo et al. 2013). However, this is likely not a concern for our study because we were not able to detect any PLIN3 or PLIN5 content in isolated sarcolemmal cuffs either at rest or with contraction. Therefore, this contamination would not be expected to alter the content of either PLIN3 or PLIN5 in our mitochondrial fraction.

## Conclusions

Mitochondrial PLIN5 content is increased during contraction, indicating a potential role for PLIN5 in communication between the fatty acids released from lipid droplets and their subsequent oxidation in the mitochondria. Further research is needed to determine whether this increased mitochondrial enrichment is due to PLIN5 movement from lipid droplets or cytosol in response to acute contraction that induced lipolysis. It is novel that PLIN3 was detected in skeletal muscle mitochondria, however, the role for PLIN3 remains elusive as mitochondrial PLIN3 content is unchanged following contraction. Future work using knockout models may be employed to determine whether mitochondrial PLIN3 protein mediates the interaction between lipid droplets and mitochondria. Our co‐immuoprecipitation data in conjunction with other data collected from our laboratory (MacPherson et al. 2013a,b) supports the idea of skeletal muscle PLIN proteins and lipases working together to regulate lipid droplet metabolism, exemplifying the complexity of lipolysis in skeletal muscle. Further work is required to determine the intracellular location of both PLIN3 and PLIN5 to determine where the protein–protein interaction occurs and how this effects mitochondrial PLIN5 enrichment. Understanding PLIN protein function in skeletal muscle is crucial in elucidating their function as intricate regulators of lipolysis in skeletal muscle lipid metabolism.

## Acknowledgments

We thank William Gittings for making the electrical wires used for the sciatic nerve stimulation and Admir Basic for performing the mechanical skinning of the plantaris muscle.

## Conflict of Interest

No conflicts of interest, financial or otherwise, are declared by the authors.
